# Vulnerable plaques in atherosclerosis: focus on angiogenesis-associated phenotypic crosstalk

**DOI:** 10.3389/fphar.2025.1737140

**Published:** 2026-01-13

**Authors:** Xin-Zheng Hou, Ying-Tian Yang, Jian-Ming Yao

**Affiliations:** 1 Department of Cardiovascular Diseases, Guang ‘anmen Hospital, China Academy of Chinese Medical Sciences, Beijing, China; 2 Department of Cardiovascular Diseases, Guang ‘anmen Hospital Jinan, China Academy of Chinese Medical Sciences, Jinan, China

**Keywords:** angiogenesis, apoptosis, atherosclerosis, extracellular matrix, inflammation, oxidative stress, vulnerable plaque

## Abstract

Atherosclerosis (AS) constitutes a major cardiovascular disorder posing a severe threat to human health, with the rupture of vulnerable plaques marking a critical turning point in the progression of AS. This pathological event can trigger acute myocardial infarction and stroke, thereby exerting a profound adverse impact on patient prognosis. In contrast to normal arterial tissues, vulnerable plaques are characterized by an abundance of neovascularization, which is generated through angiogenic pathways. Although these neovessels serve to alleviate the hypoxic microenvironment within the plaque, they concurrently compromise plaque stability. Notably, angiogenesis engages in crosstalk with AS-associated phenotypic processes, including cellular apoptosis, extracellular matrix remodeling, inflammatory responses, and oxidative stress. This interplay forms a positive feedback loop that further exacerbates the destabilization of vulnerable plaques. The vascular endothelial growth factor (VEGF) pathway plays a central regulatory role in angiogenesis. Targeting the VEGF pathway to inhibit angiogenesis and enhance plaque stability has thus opened a novel therapeutic avenue for AS management. In comparison, this strategy has demonstrated promising efficacy in preclinical studies; however, a lack of safe and reliable pharmaceutical agents remains, hindering their translation into clinical practice for AS treatment. In this review, the authors summarize the underlying mechanisms governing angiogenesis and vulnerable plaque formation, and further explore the phenotypic crosstalk between these processes. The ultimate aim is to provide valuable insights to facilitate future breakthroughs in the development of therapeutic strategies for AS.

## 1 Introduction

Atherosclerosis (AS) is a lipid-driven inflammatory disease that primarily affects large and medium-sized arteries, characterized by lipid deposition in the arterial intima and the formation of atherosclerotic plaques (Monaco et al., 2025). AS serves as the most significant pathological basis for ischemic heart disease, stroke, and peripheral arterial disease, imposing a substantial disease burden worldwide (Herrington et al., 2016; So et al., 2020; Hou et al., 2025). Vulnerable plaques refer to those atherosclerotic plaques with an increased risk of rupture and thrombosis, representing a turning point for the acute exacerbation of AS (Björkegren and Lusis, 2022). Patients with AS exhibiting features of vulnerable plaques face a significantly elevated risk of adverse events such as acute myocardial infarction and cardiac death (Del et al., 2024). Promoting the evolution of atherosclerotic plaques towards a stable phenotype is a practical approach to improving the prognosis of AS (Räber et al., 2019).

Angiogenesis refers to the process of forming new capillaries from pre-existing vascular networks, which serves as a crucial mechanism in physiological and pathological processes, such as organismal growth and development, tissue repair, and tumorigenesis (Hutchings et al., 2021). As one of the significant characteristics of vulnerable plaques, dense newly formed capillaries have been detected in vulnerable plaque tissues (Su et al., 2017; Cai et al., 2021; Gössl et al., 2010). These neovessels, originating from the vasa vasorum in the adventitia of arterial walls, while meeting the demand for blood and oxygen supply in the expanding plaque tissues, can promote inflammatory cell infiltration, cholesterol deposition, and intraplaque hemorrhage, thereby impairing plaque stability (Cheng et al., 2013; Zorc-Pleskovič et al., 2018). The inhibition of intraplaque angiogenesis provides a novel strategy for stabilizing atherosclerotic vulnerable plaques (Ugusman et al., 2024). A comprehensive understanding of the mechanisms underlying angiogenesis and the vulnerable plaques is a prerequisite for implementing targeted interventions. This review summarizes the mechanisms of angiogenesis and vulnerable plaque formation, with a focus on the phenotypic interactions between them, and reveals potential targets for regulating angiogenesis to intervene in plaque stability. Additionally, the latest advancements in interventional strategies targeting angiogenesis in vulnerable plaques will be discussed. The data for this review were retrieved from PubMed and ClinicalTrials.gov, with keywords including “atherosclerosis”, “vulnerable plaque”, “angiogenesis”, “neovascularization”, and “vascular endothelial growth factor”. Studies published between 1980 and 2025 were included, with a particular focus on those released in the recent 5 years.

## 2 Mechanisms of neovascularization

### 2.1 Vasculogenesis, arteriogenesis, and angiogenesis

**FIGURE 1:**
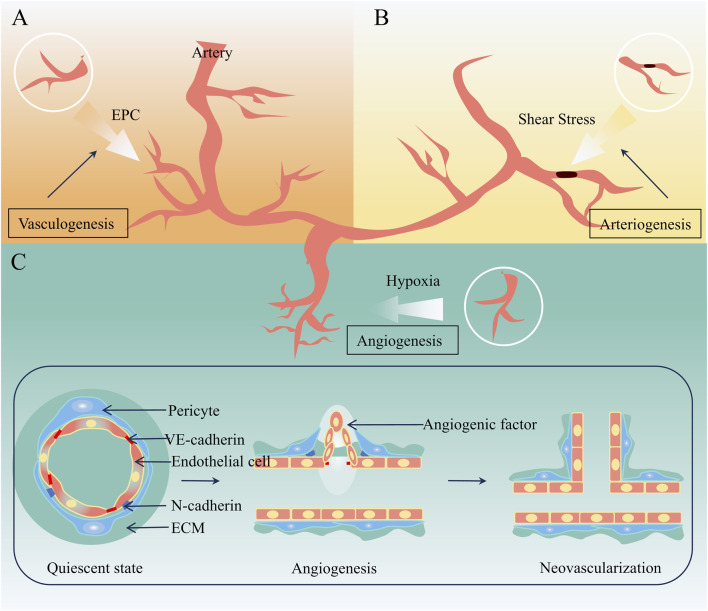
Mechanisms related to neovascularization. **(A)** Vasculogenesis **(B)** Arteriogenesis **(C)** Angiogenesis.

In physiological or pathological conditions, neovascularization occurs via vasculogenesis, arteriogenesis, and angiogenesis, each differing in initiation, process, and outcomes. Vasculogenesis refers to the formation of the primary vascular system from mesodermal progenitors during the early stages of embryonic development (Hutchings et al., 2021). Arteriogenesis, driven by hemodynamic forces, involves positive remodeling of pre-existing collateral arterioles—narrow, high-resistance vessels—into larger conductance arteries to compensate for occluded trunks (Helisch and Schaper, 2003; Moraes et al., 2013). Occurring in tissues with pre-existing collaterals, it generates structurally and functionally intact arteries, thereby restoring perfusion to ischemic regions (Wang et al., 2025). Angiogenesis, distinct from the above, involves the migration and proliferation of differentiated endothelial cells to form new capillary networks from existing vessels, encompassing sprouting and intussusceptive subtypes (Semenza, 2014). Sprouting—budding and branching from existing vessels—predominates in forming small vascular plexuses and plays a key role in neovascularization of atherosclerotic plaques (Mitsos et al., 2012). Mechanisms related to neovascularization are illustrated in Figure 1.

### 2.2 Mechanisms of angiogenesis

**FIGURE 2:**
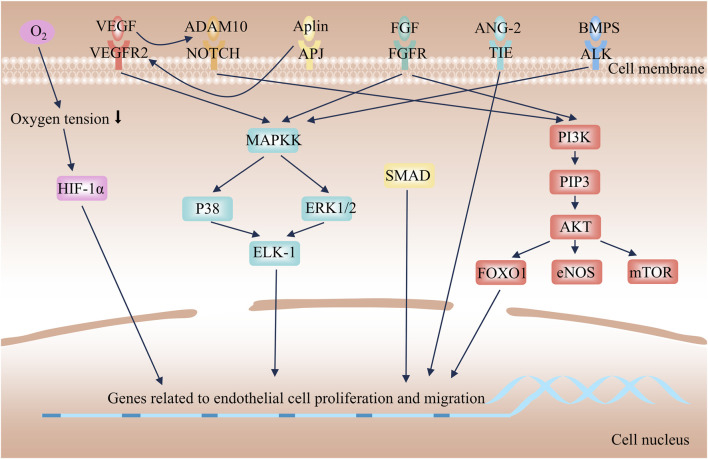
Mechanism of angiogenesis.

Mature capillaries consist of interconnected endothelial cells on the inner side and tightly arranged pericytes, along with an extracellular matrix (ECM), on the outer side, which collectively ensure normal blood circulation (Bergers and Song, 2005). Under physiological conditions, endothelial cells stay in a quiescent state, regulating blood flow to provide nutrients and oxygen for tissues. When triggers like hypoxia exist, the biosynthesis of pro-angiogenic signals increases, thereby activating angiogenesis. This process encompasses stages such as cell proliferation, migration, differentiation, maturation, and lumen formation, each featuring unique endothelial cell functions (Xiang et al., 2024). The basic process is as follows: A variety of angiogenic factors induce vascular dilation and enhance permeability by disrupting intercellular junctions and degrading the ECM. Then, endothelial cells (stalk cells and tip cells) migrate with the ECM serving as a scaffold, facilitating the formation of branches. Eventually, branch fusion, ECM deposition, and pericyte maturation result in the formation of functional new blood vessels. The molecular mechanism of angiogenesis is depicted in Figure 2. Angiogenesis is extensively involved in various physiological and pathological processes such as the AS, tumorigenesis, and tissue repair. Its purpose, regulatory mechanisms, and biological effects vary across different diseases. In brief, angiogenesis is a key driver of tumor progression and represents a pathological uncontrolled proliferation. It aims to provide nutrients and pathways for tumor growth and metastasis, with more complex regulatory mechanisms—tumor cells continuously secrete pro-angiogenic factors while inhibiting anti-angiogenic signals (Liu X. et al., 2025). Angiogenesis during wound healing is a physiological repair process with temporal specificity (Huang et al., 2025). Its goal is to restore blood supply to damaged tissues, support granulation tissue formation and epithelialization, and naturally terminate once wound healing is completed. In AS, angiogenesis is a pathological process primarily occurring within atherosclerotic plaques. While it intends to supply oxygen to hypoxic plaque tissues, it exacerbates plaque instability and rupture risk.

#### 2.2.1 Angiogenic factors

Multiple molecules mediate the process of angiogenesis. Among them, the vascular endothelial growth factor (VEGF) and the heparin - binding fibroblast growth factor (FGF) family are the core angiogenic factors. VEGF is recognized as the most critical one. The VEGF family comprises six structurally related proteins, including VEGF-A, VEGF-B, VEGF-C, VEGF-D, VEGF-E, and placental growth factor (PlGF) (Lee et al., 2015). Angiogenesis primarily relies on the interaction between VEGF-A (often referred to simply as VEGF) and VEGF Receptor-2 (VEGFR2) (Ferrara, 2002). VEGF acts as a potent vascular protective cytokine, responsible for maintaining the homeostasis of the vascular network. Simultaneously, it serves as an indispensable key component for angiogenesis in various diseases (Lee et al., 2025). Researchers have demonstrated that VEGF can induce increased vascular permeability, vasodilation, and degradation of the ECM (Choi et al., 2025; Nagasaki et al., 2016). Similarly, FGF has received extensive attention. The FGF family consists of 22 members, which bind to seven types of receptors (Phan et al., 2021). FGF-1 is referred to as acidic FGF (aFGF), and FGF-2 is known as basic FGF (bFGF); both are potent mitogens for endothelial cells (Kharitonenkov, 2009). FGF can induce *de novo* angiogenesis and plays a crucial role in the growth of new blood vessels in various scenarios, such as wound healing and embryonic development (Itoh and Ornitz, 2011).

#### 2.2.2 Angiogenic signaling pathway

Angiogenic factors are upregulated in response to stimuli such as hypoxia, activating downstream angiogenesis signaling pathways to promote endothelial cell sprouting, migration, and lumen formation. Hypoxia-inducible factors (HIFs) are a class of transcription factors activated under hypoxic conditions, playing a central role in hypoxic adaptation (Acuña-Pilarte and Koh, 2025). Tissue hypoxia inhibits the ubiquitin-proteasomal degradation of HIF-1α, allowing it to translocate into the nucleus, where it binds to the β subunit and recruits transcriptional coactivators. This complex then binds to hypoxia response elements on target genes, such as VEGF-A and FGF, inducing the expression of pro-angiogenic signaling molecules (Plastino et al., 2021; Basavaraju et al., 2021). Increased biosynthesis of pro-angiogenic signals induces matrix metalloproteinases (MMPs) to degrade ECM components. This process leads to the detachment of pericytes from the basement membrane, disruption of endothelial junctions, and increased vascular permeability, thereby creating conditions that are conducive to endothelial cell migration (Carmeliet and Jain, 2011). Individual endothelial cells, known as tip cells, undergo sprouting and migration in response to pro-angiogenic signaling molecules. The migration of tip cells relies on PI3K/Akt-eNOS-mTOR-FOXO1 axis-mediated cell proliferation and cytoskeletal remodeling (Ackah et al., 2005; Fukumura et al., 2001). Additionally, the FGF/FGFR and Apelin/APJ systems synergistically promote this process by regulating energy metabolism and directional polarity (Vimalraj, 2022; Wagenaar and Moll, 2025). Concurrently, BMP2 enhances sprouting through the activation of the PI3K/Akt and MEK/ERK pathways (Cai et al., 2012; Goumans et al., 2009; Chen et al., 2018). Notch signaling further activates the PI3K/Akt pathway to sustain the survival and proliferation of endothelial cells (Gude et al., 2008; Meurette et al., 2009). The aforementioned signaling pathways collectively synergize to promote the sprouting and migration of endothelial cells. Following the tip cells are endothelial cells called stalk cells, which are responsible for lumen elongation and pericyte recruitment (ávila-Gálvez et al., 2025). After branch fusion, endothelial cells in the new blood vessels revert to a quiescent state, reorganize cellular junctions, promote pericyte maturation, deposit the basement membrane, and maintain the balance between pro-angiogenic and anti-angiogenic factors (Liu et al., 2023).

#### 2.2.3 Emerging detection technologies for angiogenesis

A variety of imaging technologies can achieve quantitative analysis of angiogenesis and neovascularization by virtue of different biomarkers. With the continuous emergence of emerging techniques such as single-cell RNA sequencing, spatial transcriptomics, and high-resolution imaging of vasa vasorum, more precise methods have been provided for angiogenesis detection. Most studies adopt immunohistochemical staining or immunofluorescence staining to evaluate neovascularization, and the biomarkers used include CD31, CD34, vWF, Ki67, and TER-119 (Parma et al., 2020; Tziakas et al., 2013; Kurdi et al., 2019; Qi et al., 2023). All the aforementioned methods are indirect detection approaches. Contrast-enhanced ultrasound and micro-ultrasound, two non-invasive techniques for neovascularization detection, enable dynamic imaging of blood flow status (Liu et al., 2017; Ding et al., 2019). The application of single-cell RNA sequencing facilitates the accurate identification of specific cell subsets and specific molecular expressions (Zhao et al., 2025). Spatial transcriptomics overcomes the limitation of traditional sequencing in losing spatial location information. It can resolve gene expression profiles while preserving the morphological characteristics of tissues (Liu S. et al., 2025). High-resolution imaging of vasa vasorum is a pivotal technique for the precise detection of angiogenesis from a morphological perspective. It can compensate for the limitations of traditional molecular detection and conventional imaging techniques, and intuitively visualize the morphological characteristics and distribution patterns of vasa vasorum (Cebral et al., 2025).

## 3 Mechanism of vulnerable plaque

**FIGURE 3:**
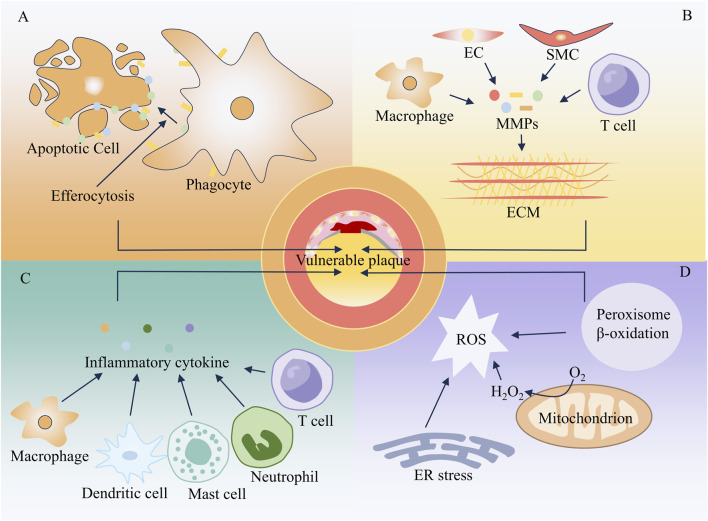
Mechanism of vulnerable plaque formation. **(A)** Apoptosis **(B)** Extracellular matrix remodeling **(C)** Inflammatory response **(D)** Oxidative stress.

With the progression of AS, the arterial intima may develop intimal thickening, fatty streaks, fibrolipid plaques, calcified plaques, or complex plaques. Plaque rupture, surface erosion, and calcified nodules are the primary pathological bases for acute thrombosis in AS, with plaque rupture playing a dominant role (Yahagi et al., 2016). Plaques with a high tendency to rupture are defined as vulnerable plaques, and the most common morphological type is the thin-cap fibroatheroma (TCFA) (Fleg et al., 2012). TCFA exhibits typical phenotypic characteristics: it contains a large necrotic core composed of lipids and cellular debris, covered by a thin fibrous cap (usually less than 65 μm in thickness) (Suzuki et al., 2024; Roleder-Dylewska et al., 2023). A large number of inflammatory cells infiltrate the fibrous cap, often presenting with punctate calcification and intraplaque hemorrhage. Additionally, the arterial wall may undergo aneurysmal dilatation and remodeling. As shown in Figure 3, cell apoptosis, impaired phagocytosis, ECM remodeling, inflammatory responses, and oxidative stress are closely associated with the formation of TCFA-type vulnerable plaques.

AS can affect systemic arterial vessels such as the coronary arteries and peripheral arteries. Distinct disparities exist in the regulatory mechanisms governing plaque stability across different vascular territories, with the differentiation of angiogenesis patterns serving as the core demarcation. Coronary arteries are characterized by abundant bifurcations, low blood flow shear stress and prominent turbulent flow, which lead to a high degree of hypoxia within the plaques. This robustly activates the VEGF pathway, driving extensive proliferation of adventitial vasa vasorum and subintimal vascular plexuses. The resultant neovessels feature high density and diffuse distribution, markedly elevating the risk of plaque rupture. In contrast, peripheral arteries exhibit relatively stable blood flow shear stress and milder plaque hypoxia. Neovascularization in peripheral arterial plaques is mostly confined to the adventitia, with a density merely half that observed in coronary plaques. Additionally, peripheral arterial plaques display significant calcification and more uniform plaque stress distribution. Consequently, these plaques possess substantially greater overall stability. These differences provide a critical theoretical basis for the clinical implementation of targeted interventions against vulnerable plaques.

### 3.1 Cell apoptosis and efferocytosis impairment

One of the characteristics of vulnerable plaques is a large necrotic core covered by a thin fibrous cap, and the apoptosis of smooth muscle cells (SMCs) and macrophages is the leading cause (Silvestre-Roig et al., 2014; Yin et al., 2024). SMCs are the primary component of the fibrous cap, promoting its thickening by synthesizing matrix components, including collagen and elastin. In the fibrous cap of vulnerable plaques, the apoptosis of SMCs is exacerbated, which reduces the synthesis of matrix proteins—contributing to the formation of a thin fibrous cap and the expansion of the necrotic core (Rotllan et al., 2015). The apoptosis of SMCs is mainly induced by the continuous infiltration of inflammatory cells and protease-mediated changes in the extracellular environment. Additionally, the accumulation of degradation products from the fibrous cap matrix can also induce SMC apoptosis (von Wnuck et al., 2006). Macrophage apoptosis occurs in all stages of AS. In the early stage, it helps reduce plaque burden; however, in the late stage, it impairs plaque stability (Deng et al., 2025). As the disease progresses, a large number of apoptotic macrophages cannot be cleared promptly and accumulate in the plaque, leading to the expansion of the necrotic core and exacerbation of local inflammatory responses (Gautier et al., 2009). In the late stage of AS, the accumulation of free cholesterol (caused by impaired esterification function) and exposure to oxidized cholesterol trigger the unfolded protein response (UPR) (Moore and Tabas, 2011). When the UPR is chronically activated, it induces macrophage apoptosis (Thorp et al., 2009). Furthermore, impairment of macrophage autophagy can also induce excessive apoptosis (Liao et al., 2012).

When efferocytosis is impaired, these protective functions are weakened, thereby contributing to the development of AS (Schrijvers et al., 2005). Efferocytosis refers to the process by which phagocytes clear apoptotic cells, thereby preventing the accumulation of apoptotic cell debris and triggering the reprogramming of phagocytes toward an anti-inflammatory phenotype. In the early stages of AS, dying cells can be rapidly cleared; however, as the disease progresses, efferocytosis becomes defective (Yurdagul, 2021). Since chemotactic and phagocytic signals are usually abundant in advanced plaques, the defect in efferocytosis may result from impaired phagocytic function of macrophages within the lesion, rather than a lack of signals that attract phagocytes.

### 3.2 Remodeling of ECM

ECM remodeling constitutes an indispensable step in the formation of vulnerable atherosclerotic plaques. The ECM is a complex reticular scaffold structure composed of collagen fibers, elastin, fibronectin, laminin, and other components. These components are distributed in the basement membrane or interstitial matrix, providing structural support for tissues. In the early stage of AS, the ECM consists of laminin, polymerized type Ⅰ collagen, type Ⅲ collagen, type Ⅳ collagen, a small amount of fibronectin, and elastin fibers. In the late stage of AS, the ECM comprises monomeric type I collagen, fibronectin, fibrinogen, vitronectin, and osteopontin (Yurdagul et al., 2016; Ström et al., 2004). ECM remodeling affects plaque stability primarily by regulating the mechanical strength of the fibrous cap and vascular function. Collagen is the main component of the ECM and is particularly abundant in atherosclerotic plaques, among which type Ⅰ collagen accounts for the highest proportion (approximately 60%) (Holm et al., 2019). Studies on sirtuin 1 (SIRT1) have confirmed the importance of type Ⅰ collagen in plaque stability. Specifically, SIRT1 deacetylates regulatory factor X5 (RFX5), thereby relieving the inhibitory effect of RFX5 on the promoter activity of the collagen α2 chain (Xia et al., 2012). This process increases the expression of type Ⅰ collagen and prevents plaque rupture. Elastin enables blood vessels to adapt to changes in hemodynamics. In the intima of healthy arteries, the content of soluble elastin is high; however, its content decreases in ruptured plaques (Maedeker et al., 2016). A study has verified that inhibiting microRNA-181b can significantly increase elastin expression and stabilize plaques (Di Gregoli et al., 2017).

ECM remodeling is primarily driven by proteases, which represent a key process leading to the reduced mechanical strength of the fibrous cap (Newby, 2012). MMPs are a family of zinc-dependent endopeptidases that can degrade almost all other ECM components (Galis and Khatri, 2002; Li et al., 2014). In vulnerable plaques, in addition to endothelial cells, T cells, macrophages, and SMC can all increase the production of MMPs (Bäck et al., 2010). In AS, the expression of MMP-1 and MMP-8 is significantly upregulated, and their levels are even higher in vulnerable plaques (Molloy et al., 2004; Müller et al., 2014). As collagenases, MMP-1 and MMP-8 can degrade fibrillar collagen (Libby, 2013). In a model of AS with MMP-8 knockout, the plaque area was significantly reduced (Laxton et al., 2009). MMP-2 and MMP-9, as major gelatinases, can degrade a variety of ECM proteins, including collagen (Siefert and Sarkar, 2012). Compared with stable plaques, MMP-2 and MMP-9 in vulnerable plaques are 20 times higher (Guo ZY. et al., 2018). The aforementioned studies indicate that MMPs can regulate plaque stability by remodeling the ECM.

### 3.3 Inflammatory response

Inflammation plays a crucial role in plaque rupture, and vulnerable plaques exhibit obvious signs of inflammation (Aukrust et al., 2011). During the progression of AS, macrophages, dendritic cells (DCs), and mast cells can affect plaque stability through phenotypic changes and the secretion of cytokines. An extensive cohort study involving 200 human carotid plaques reported an association between CD163^+^ macrophages and the vulnerable plaque phenotype (Bengtsson et al., 2020). In vulnerable plaques, the number of mature dendritic cells is significantly increased. Due to the interaction between dendritic cells and regulatory T cells (Tregs), the migration of DCs and their adhesion to endothelial cells are directly inhibited, thereby impairing plaque stability (Dietel et al., 2013). The frequency of CD117^+^ mast cells is higher in unstable plaques than in stable ones (Joo et al., 2020). Mast cells are activated by binding to IgE attached to the Fc epsilon receptor (FcεR), which leads to the release of cytoplasmic granules containing proinflammatory factors, histamine, and proteases (Kritikou et al., 2019). As a crucial driver of AS, the understanding of inflammatory responses has been progressively deepened. Some researchers have proposed an inflammation-based 3-category classification of atherosclerotic plaques, namely active plaques, dormant plaques, and inactive plaques, analogous to the activity states of volcanoes (Miceli et al., 2025). Even clinically “stable” plaques may exhibit silent yet persistent immunometabolic and thromboinflammatory activities, thereby elevating residual cardiovascular risk. This evolving paradigm endorses immunomodulation as the cornerstone of precision cardiovascular medicine.

### 3.4 Oxidative stress

Oxidative stress serves as a vital initiating and promoting factor in the development of vulnerable plaques. Oxidative stress refers to a state where the body, upon exposure to various stimuli, generates excessive amounts of highly reactive molecules. When the degree of oxidation exceeds the scavenging capacity of antioxidants, an imbalance occurs between the oxidative and antioxidant systems, leading to tissue damage. Reactive oxygen species (ROS) are a group of oxygen-containing molecules or oxygen atoms with unpaired electrons, including superoxide anions, hydroxyl radicals, and hydrogen peroxide. Among these, superoxide anions are produced in the largest quantity. Studies have shown that the size of AS lesions is closely correlated with the rate of ROS production; inhibiting oxidative stress and ROS generation can suppress the formation of vulnerable plaques (Zhu et al., 2024). *In vitro* studies have shown that inhibiting ROS production can effectively prevent macrophage apoptosis, thereby reducing the risk of atherosclerotic plaque rupture (Fang et al., 2021). *In vivo* studies have demonstrated that targeted scavenging of ROS in AS model mice using nanoparticle therapy can significantly inhibit AS progression, providing a novel approach for the diagnosis and treatment of vulnerable plaques (Dai et al., 2022). Key catalytic enzymes involved in oxidative stress include nicotinamide adenine dinucleotide phosphate (NADPH) oxidase, xanthine oxidase, and endothelial nitric oxide synthase (NOS). In human atherosclerotic plaques, superoxide anions and their leading producers—NADPH oxidase and xanthine oxidase—are present in large quantities (Förstermann et al., 2017). Inhibiting nitric oxide (NO) derived from NOS is an effective strategy for reducing foam cell formation and limiting the progression of atherosclerotic plaques (Roy et al., 2021).

## 4 Angiogenesis and vulnerable plaques

**FIGURE 4:**
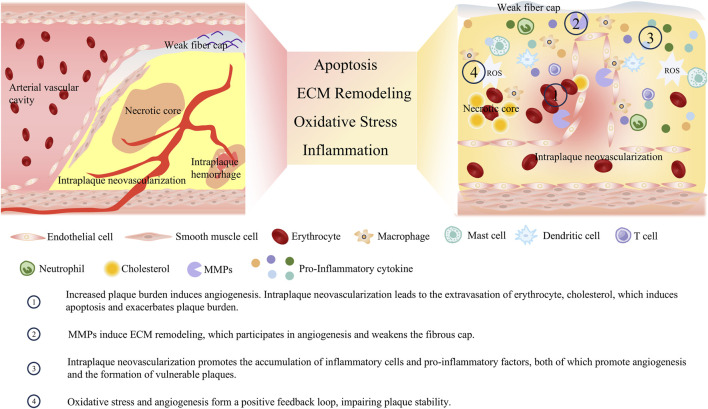
Crosstalk between angiogenesis and vulnerable plaque phenotype.

Vasa vasorum originates from the branching points of arteries, extends longitudinally along the vascular wall, and subsequently forms circular arches surrounding the arterial lumen (Svetlikov et al., 2025). Since the diffusion of blood-borne nutrients from the central arterial lumen is limited to a distance of approximately 100 μm, the primary function of the vasa vasorum is to transport nutrients to the vascular wall (Cebral et al., 2025). Angiogenesis exerts distinct effects at different stages of atherosclerotic lesions: it primarily functions in tissue repair during the early phase but becomes a major pathogenic factor in the advanced stage. In the early stage of the AS, angiogenesis improves blood supply to ischemic regions, alleviates hypoxic injury to vascular endothelial cells, and delays the progression of endothelial dysfunction. As plaques advance, the newly formed blood vessels exhibit fragile structures that are prone to rupture and hemorrhage, triggering intraplaque hematoma and accelerating plaque instability or even rupture. In the advanced stage, the high permeability of neovessels increases the leakage of plasma proteins and lipids, further exacerbating the inflammatory response and lipid deposition within plaques, promoting plaque enlargement and lipid core expansion, and aggravating arterial stenosis. As AS progresses, intimal thickening causes the vascular wall thickness to exceed the critical threshold distance of 100 μm. This exceeds the limit of oxygen and nutrient supply to the media and hyperplastic intima, creating a hypoxic environment within the artery. Hypoxia further activates angiogenesis (Giannarelli et al., 2014). This activation of angiogenesis aims to improve the oxygen and nutrient supply to vascular tissues, serving as a permissive factor that allows plaque growth to continue after the critical intimal thickness is reached (Pan et al., 2024). While intraplaque microvessels contribute to compensatory blood supply, they are closely associated with plaque rupture. Compared with unruptured plaques, ruptured plaques exhibit increased microvessel density—this increase is particularly prominent in the shoulder regions of plaques. These findings suggest that angiogenesis is involved in the formation of vulnerable plaques and may serve as a key target for regulating plaque stability (Virmani et al., 2005). As presented in Figure 4, numerous studies have explored the role of angiogenesis in the development of vulnerable plaques.

### 4.1 Angiogenesis and plaque burden

Angiogenesis is an inevitable consequence of the progression of plaque burden. Meanwhile, the immature neovessels can induce intraplaque hemorrhage, expand the necrotic core, and exacerbate plaque burden. As mentioned earlier, the hypoxic microenvironment within the large necrotic core of vulnerable plaques can activate angiogenic pathways. While the neovessels formed via angiogenic pathways provide oxygen and nutrients to the plaque, they also facilitate the influx of lipids, leukocytes, and red blood cells. This influx leads to the expansion of the necrotic core and intraplaque hemorrhage, which increases the plaque burden and impairs plaque stability (Moreno et al., 2012; Kwon et al., 2015). Intraplaque hemorrhage is a critical determinant of plaque instability and is closely associated with the occurrence of future ischemic events (Faghani et al., 2025). It accelerates plaque progression by increasing the size of the necrotic core and the overall plaque volume, resulting from the accumulation of lipid-rich red blood cell membranes within the plaque (Zhao et al., 2022). Such hemorrhage originates from the rupture and leakage of structurally immature neovessels. Studies have found that 80% of intraplaque neovessels exhibit poor integrity, with loose endothelial junctions, insufficient pericyte coverage, and a lack of SMC support, making them prone to leakage (Sluimer et al., 2009). In human carotid plaques, macrophages are typically found surrounding hemorrhagic areas. These macrophages participate in the phagocytosis of red blood cells and iron, and can also secrete VEGF, further enhancing the permeability of neovessels (Guo L. et al., 2018). During the leakage process, blood cells infiltrate into the plaque core, and their cell membranes release free cholesterol. This free cholesterol promotes the formation of cholesterol crystals, which can erode the fibrous cap and protrude into the arterial lumen, potentially triggering embolism or thrombosis (Abela et al., 2009). Undoubtedly, the aforementioned blood cells, cholesterol, and other substances extravasated through immature neovessels further increase the burden on phagocytes within the plaque, exacerbate macrophage apoptosis, and augment plaque burden.

### 4.2 Angiogenesis and ECM remodeling

Both angiogenesis and plaque tissue matrix remodeling rely on the regulation of MMPs, making MMPs the mediator that bridges these processes. As previously mentioned, a variety of MMPs are involved in the formation of vulnerable plaques; they also act as driving factors for angiogenesis, while neovessels can, in turn, accelerate vascular remodeling by affecting MMPs. A range of angiogenic factors can induce the activation of MMPs. Stimulated by VEGF and bFGF, endothelial cells secrete vesicles containing MMP-2 and MMP-9, thereby regulating tip cell migration and lumen formation (Taraboletti et al., 2002). After inhibiting the expression of MMP-2, the proliferation and migration capabilities of endothelial cells decrease, and the growth of the new capillary network is suppressed (Cheng et al., 2007). MMP-1 can promote the expression of VEGFR2 and the proliferation of endothelial cells; it exerts this effect by stimulating serine/threonine protein kinases and activating the transcription factor nuclear factor-κB (Mazor et al., 2013). MMP-7 can regulate the VEGF pathway and indirectly promote angiogenesis by degrading soluble VEGFR1 (Libby et al., 2011; Ito et al., 2009). The aforementioned MMPs involved in angiogenesis can accelerate the degradation of the fibrous cap, thereby exacerbating plaque instability or rupture (Leclercq et al., 2007). At the same time, cells that extravasate through immature neovessels can release TNF-α, IL-8, and other cytokines, which further stimulate endothelial cells to produce MMP-2, MMP-8, and MMP-9, thereby regulating the processes of angiogenesis and vascular remodeling (Li et al., 2005; Akbari et al., 2023).

### 4.3 Angiogenesis and inflammatory response

Inflammation and angiogenesis in vulnerable plaques are interconnected. On the one hand, various inflammatory cells infiltrate the plaque and secrete inflammatory and growth factors, which promote the formation of new blood vessels. On the other hand, neovessels can facilitate the infiltration of blood components, such as leukocytes, into the plaque, thereby exacerbating the inflammatory response. This positive feedback loop impairs plaque stability, intensifies inflammation, and leads to the formation of more defective neovessels. Inflammation increases the local metabolic rate, characterized by elevated glucose uptake and oxygen consumption, which further aggravates local hypoxia. Hypoxia serves as a crucial stimulus for angiogenesis. A hallmark of plaques is the infiltration of inflammatory cells, such as macrophages. These cells can secrete VEGF and bFGF, actively participating in angiogenesis. Additionally, inflammatory cells can secrete classical inflammatory cytokines with pro-angiogenic functions, including IL-6, TNF-α, MCP-1, and IL-8. Beyond the aforementioned classical pro-angiogenic factors, inflammatory cells can secrete specific factors that promote angiogenesis. For instance, the macrophage-derived peptide PR39 can inhibit the ubiquitin-proteasome-dependent degradation of the HIF-1α, thereby accelerating the formation of vascular structures (Li et al., 2000). Numerous pro-angiogenic factors, in turn, can exacerbate the inflammatory response. VEGF can stimulate the migration of monocytes into the plaque (Chen et al., 2025). In hypercholesterolemic mice, infusion of low-dose VEGF increases the recruitment of monocytes from the bone marrow to the bloodstream, ultimately exacerbating the plaque burden (Celletti et al., 2001). bFGF can synergize with inflammatory factors, including TNF-α, to enhance the expression of adhesion molecules on activated endothelial cells, thereby promoting leukocyte recruitment (Zittermann and Issekutz, 2006). Intraplaque hemorrhage secondary to neovessel formation can activate platelets and promote the retention of leukocytes on the vascular endothelium, thereby further exacerbating inflammation (May et al., 2008).

Recent studies have highlighted the role of neutrophil extracellular traps (NETs) in neovascularization. In mouse models of choroidal neovascularization, the levels of NETs are elevated, and inhibition of NETs can significantly reduce the formation of new blood vessels. *In vitro*, NETs are capable of stimulating the proliferation, migration and tube formation of endothelial cells. The underlying mechanism involves the activation of endothelial cells via the TLR4/HIF-1α pathway, which in turn leads to the upregulation of MMP-9 and IL-1β—both of which are key mediators of angiogenesis (Zeng et al., 2023). The involvement of NETs in angiogenesis has been verified in various diseases such as tumors and pulmonary arterial hypertension (Yang et al., 2023; Aldabbous et al., 2016).

### 4.4 Angiogenesis and oxidative stress

Oxidative stress and angiogenesis together accelerate the formation of vulnerable plaques. The interaction between oxidative stress and angiogenesis centers on the VEGF signaling pathway. ROS generated during arterial endothelial injury upregulate VEGF expression; for example, hydrogen peroxide can induce VEGF expression in both SMCs and endothelial cells, thereby promoting angiogenesis (Bretón-Romero and Lamas, 2014). Additionally, after endothelial cells are stimulated by ox-LDL, they produce ROS that activate angiogenesis-related signaling pathways, including those involving p38 mitogen-activated protein kinase (p38MAPK) and extracellular regulated protein kinases 1/2 (ERK1/2). Antioxidants can block the activation of these aforementioned signaling pathways, thereby inhibiting angiogenesis (Dandapat et al., 2007; Camaré et al., 2015; Kim and Byzova, 2014). ROS also affect the activation, aggregation, and phosphorylation of VEGFR2 (Colavitti et al., 2002). Furthermore, oxidized products, such as oxidized phospholipids have been shown to interact with VEGFR2 and activate angiogenesis (Birukova et al., 2012). In summary, oxidative stress promotes angiogenesis by acting on upstream and downstream components of the VEGF/VEGFR2 signaling pathway. Conversely, intraplaque angiogenesis can further activate oxidative stress. Microhemorrhage caused by intraplaque neovessels is directly associated with oxidative stress (Yunoki et al., 2009; Nagy et al., 2010). Hemorrhage from immature neovessels releases hemoglobin and iron; these substances can promote the production of free radicals and ROS (e.g., via the Fenton reaction), which in turn inactivate nitric oxide and drive lipid peroxidation (Camaré et al., 2017). Additionally, VEGF can activate NADPH oxidase in endothelial cells, thereby promoting ROS generation (Huang and Nan, 2019).

## 5 Anti-angiogenic therapy in AS

Given the critical role of angiogenesis in the formation of vulnerable plaques, a large body of research has focused on this field, and preclinical studies have demonstrated promising application prospects. The number of compounds targeting VEGF and its upstream/downstream pathways to inhibit angiogenesis has increased significantly, opening up a new research direction for the treatment of AS. In the long term, using intraplaque angiogenesis inhibitors as an adjuvant therapy to statins for plaque stabilization has emerged as a potential research avenue.

Preclinical studies have demonstrated that antagonizing key pro-angiogenic factors and regulating endothelial cell metabolism are promising strategies for anti-angiogenesis. Among these, the antagonism of key pro-angiogenic factors has been the most extensively studied. In murine models, anti-VEGF compounds, such as endostatin and fumagillin, have been shown to alleviate AS and inhibit macrophage infiltration (Moulton et al., 1999; Griffith et al., 1998). Endostar, a recombinant human endostatin, is a broad-spectrum anti-angiogenic agent that can interfere with the pro-angiogenic effects of bFGF and VEGF (17). In recent years, researchers have observed that Endostar reduces vasa vasorum formation, vascular wall inflammatory responses, and AS progression in porcine AS models (Xu et al., 2015). In hypercholesterolemic rabbits, targeted delivery of fumagillin-loaded nanoparticles effectively blocks endothelial cell proliferation and reduces intraplaque neovascularization (Winter et al., 2006). Bevacizumab, an anti-VEGF monoclonal antibody, is a potent angiogenesis inhibitor that can suppress intraplaque neovascularization and reduce the volume of AS plaques (Stefanadis et al., 2007). In murine models, bevacizumab restores pericyte function by inducing the expression of platelet-derived growth factor-B, thereby reducing vascular leakage (Xiao et al., 2016). In New Zealand rabbit models, the implantation of bevacizumab-eluting stents in the iliac artery inhibits neovascularization without impairing endothelial repair (Wang et al., 2011). Axitinib is a potent and selective inhibitor of VEGFR tyrosine kinases. In AS mice, continuous intraperitoneal injection of axitinib for 6 weeks significantly reduces intraplaque neovascularization by 50% and decreases the incidence of intraplaque hemorrhage (Van der Veken et al., 2018). Additionally, animals in the axitinib-treated group exhibit improved cardiac function and a lower incidence of myocardial infarction. By targeting VEGFR, axitinib reduces intraplaque neovascularization and hemorrhage, and promotes plaque stabilization. Beyond antagonizing VEGF-related pathways, antagonists of other pro-angiogenic factors hold developmental potential. Statins, as foundational drugs for AS treatment, have been found to inhibit angiogenesis. In animal models of hypercholesterolemia, statins inhibit neovascularization (Wilson et al., 2002; Tian et al., 2013). In addition to antagonizing pro-angiogenic factors, regulating cellular metabolism has shown beneficial effects in cancer research, and this strategy holds value in AS. Endothelial cells under hypoxic conditions exhibit high glycolytic activity, with the ATP produced accounting for more than 85% of the total cellular ATP content (De Bock et al., 2013). In glycolysis, the conversion of fructose-6-phosphate to fructose-2,6-bisphosphate, which is regulated by 6-phosphofructo-2-kinase/fructose-2,6-bisphosphatase (PFKFBs), is one of the rate-limiting steps. Intraperitoneal injection of the small-molecule compound 3PO reduces capillary sprouting by inhibiting endothelial cell proliferation and migration (Schoors et al., 2014). Studies suggest that 3PO exerts its effects by blocking PFKFB3. The inhibition is partial (35%–40%) and transient, yet sufficient to reduce neovascularization. This characteristic confers potential safety, as complete and permanent inhibition of glycolysis could lead to ATP depletion and subsequent cytotoxicity (Granchi and Minutolo, 2012). Inhibiting carnitine palmitoyltransferase 1a (CPT1a), the rate-limiting enzyme in fatty acid oxidation (FAO), also reduces endothelial cell proliferation and inhibits vascular sprouting (Schoors et al., 2015). Studies have found that etomoxir, an irreversible inhibitor of CPT1a, inhibits vascular sprouting and exhibits favorable inhibitory effects on pathological angiogenesis in ocular disease models (Missiaen et al., 2017).

Although preclinical studies have brought new hope for anti-angiogenic therapy in the treatment of AS, there is still a lack of novel anti-angiogenic agents with no unacceptable adverse reactions for clinical application in AS treatment. This may be attributed to the dual role of VEGF in maintaining arterial function. Briefly, the action of VEGF exhibits distinct concentration dependence and spatiotemporal specificity. Under pathological conditions, as mentioned earlier, it contributes to the formation of vulnerable atherosclerotic plaques. In contrast, at physiological levels, VEGF can maintain plaque stability and delay lesion progression through multiple mechanisms: promoting the repair and regeneration of endothelial cells, improving local blood supply to plaques to reduce the formation of necrotic cores, and inhibiting the excessive proliferation of vascular smooth muscle cells (Dabravolski et al., 2022). Several clinical studies have investigated the effect of anti-angiogenic agents on AS. Studies on the use of anti-VEGF drugs in cancer treatment have reported hypertension as an adverse reaction (Versmissen et al., 2019). Another study indicated that anti-VEGF therapy can induce proteinuria, leading to renal injury in kidney transplant recipients (Cheungpasitporn et al., 2015). Beyond its role in angiogenesis, the VEGF signaling pathway is involved in multiple processes, including compensation and remodeling following myocardial injury. Anti-angiogenic therapy may cause endothelial dysfunction, reduce microvascular density, and trigger adverse reactions, including hypertension, myocardial ischemia, cardiomyopathy, thromboembolic diseases, and thrombotic microangiopathy (Xiao et al., 2016; Maurea et al., 2016). For instance, a meta-analysis focusing on the use of VEGFR inhibitors in cancer treatment demonstrated an increased risk of arterial thromboembolism (Choueiri et al., 2010). However, as mentioned earlier, statins can reduce the formation of neovascularization within atherosclerotic plaques (Koutouzis et al., 2007). Therefore, the potential harmful effects of long-term anti-angiogenic therapy may not constitute a major concern, and this issue remains to be further demonstrated.

## 6 Conclusion

Intraplaque angiogenesis is a consequence of the hypoxic environment in the hyperplastic intima induced by the progression of AS. It involves a series of complex processes, including endothelial cell sprouting, proliferation, migration, as well as lumen formation and maturation, among which the activation of VEGF-related pro-angiogenic signaling plays a dominant role. While intraplaque neovessels compensate for the loss of oxygen and nutrients to the intima, these neovessels exhibit loose endothelial junctions and insufficient pericyte coverage, making them highly prone to leakage. This leakage creates conditions for the entry of cholesterol, inflammatory cells, red blood cells, ECM, and other atherogenic molecules into the plaque. In turn, this further activates phenotypes associated with vulnerable plaque formation, such as cell apoptosis, ECM remodeling, inflammatory response, and oxidative stress.

Inhibiting intraplaque angiogenesis represents a highly promising novel pharmacological target, with the potential to stabilize vulnerable plaques. In the long term, this intervention strategy may lead to the development of new therapeutic approaches for patients who cannot derive sufficient benefits from existing lipid-lowering therapies. Although numerous studies have reported an association between angiogenesis and plaque stability, pharmacological interventions targeting this phenotype are still under exploration. In experimental animal models, angiogenesis inhibitors have demonstrated efficacy in delaying AS progression and stabilizing plaques. However, clinical trial results indicate that currently available anti-angiogenic drugs carry certain risks. Anti-angiogenic drugs applicable for AS treatment remain to be developed. Among these, inhibitors targeting pro-angiogenic factor pathways, as well as inhibitors of endothelial cell glycolysis and fatty acid oxidation pathways, show considerable promise.
